# The Clinical Features and Prognosis of Gastric Remnant Carcinoma after Treatment

**DOI:** 10.5402/2011/708215

**Published:** 2011-10-30

**Authors:** Ken-Sheng Cheng, Hui-Ling Tang, Jen-Wei Chou, Cheng-Ju Yu, Shi-Seng Tsou, Fu-Tsan Chou

**Affiliations:** Department of Internal Medicine, College of Medicine, China Medical University Hospital and China Medical University, No. 2 Yu-Der Road, Taichung 40421, Taiwan

## Abstract

*Introduction*. The incidence of gastric remnant carcinoma does not decrease after partial gastrectomy. The aim of this study was to evaluate the clinical features and prognosis of gastric remnant carcinoma after treatment. *Methods*. Among 412 gastric carcinoma patients who were admitted to our hospital, 21 were found to have gastric remnant carcinoma. We analyzed their clinicopathological features and prognosis. *Results*. Prognosis did not differ significantly in terms of gender, age, tumor lymph node metastasis stage, tumor location, and time interval between first and subsequent operations. However, it was influenced by intensive curative gastrectomy with resection of local lymph nodes. *Conclusion*. Long-term follow-up after gastrectomy, appropriate curative resection, and prevention and management of comorbidities are important to detect gastric remnant carcinoma at an early stage.

## 1. Introduction

Balfour was the first to demonstrate the existence of gastric remnant carcinoma that occurred more than 5 years after partial gastrectomy for benign gastric ulcer disease. Although proton-pump inhibitor therapy and therapeutic endoscopy are currently being performed instead of partial gastrectomy for bleeding peptic ulcers, the incidence of gastric remnant carcinoma is not decreasing. This malignancy has also been described in patients who have undergone gastric bypass surgery for obesity. The causes for remnant carcinoma possibly involve gastroduodenal reflux of bile and pancreatic juice, denervation during gastrectomy with hypochlorhydria, and a long latency period. *Helicobacter pylori *infection is not an important factor in this case [1, 2].

Many studies have shown differences between the clinicopathological features of gastric remnant carcinoma and patient outcomes after partial gastrectomy for peptic ulcer complications [3–5]. The aim of this study was to evaluate the clinical features and prognosis of gastric remnant carcinoma after treatment.

## 2. Patients and Methods

Between January 2004 and November 2008, 412 patients with gastric carcinoma were admitted to our hospital. Among them, 21 were shown to have gastric remnant carcinoma after partial gastrectomy for peptic ulcers. The gender distribution in this study was 15 men and 6 women. The average age at the time of diagnosis was 66.2 years (45–85 years). All patients underwent abdominal computed tomography for staging or surgical evaluation. Age, gender, time interval between initial gastrectomy and diagnosis of cancer, and chief presentation symptoms at admission were obtained from charts of the patients. The survival time was estimated on the basis of evaluation in the first year and followed up for 4 years. The tumors were classified by site as stump, nonstump, or diffuse remnant and staged using the tumor lymph node metastasis (TNM) classification after abdominal computed tomography with or without the surgical findings. All procedures were performed under the supervision of the hospital ethics committee.

Statistical analysis was performed using Fisher's exact test. *P* < 0.05 was considered statistically significant.

## 3. Results

Among the 21 patients, only 2 had undergone Billroth I gastrectomy. The mean interval between the first surgery and diagnosis of remnant gastric carcinoma was 30.5 years (12–55 years). The presented symptoms were epigastric pain and fullness, followed in order by tarry stools or hematemesis, vomiting, and poor appetite (Table 1). 

Nine patients survived for over 2 years; 8 had undergone total gastrectomy with or without lymph node resection, and 1 had been treated with chemotherapy (5-fluorouracil). Tumors in all these patients were located at the remnant of the gastric tract (Table 2). The mean tumor size was 3.5 ± 2.0 cm. Among the 12 patients who survived for less than a year, resection of lymph nodes was not performed and distant metastasis was not observed, or in some case, they had comorbidities such as cardiovascular disease (4 patients had unstable hypertension, myocardial ischemia arrhythmia, uremia, or cerebral stroke). The tumors were located at the remnant site in 7 patients, at the nonremnant site in 2, and at a distant site in 3 (Table 3); the mean tumor size was 4.5 ± 3.0 cm.

Survival time did not differ significantly in terms of gender, age, TNM stage, tumor location, or time interval between initial and subsequent operations (*P* > 0.05). The only factor that influenced survival was intensive curative gastrectomy with resection of local lymph nodes, as illustrated in Tables 2 and 3.

## 4. Discussion

The incidence of gastric remnant carcinoma after sub-total gastrectomy, especially via the Billroth I operation, varies from 0.4% to 5%, and the cumulative risk correlates with increased incidence 15 years after the operation. The overall 5-year disease-free survival is reportedly between 7% and 20%. A low incidence of gastric remnant carcinoma was observed in the Billroth I operation, and a remnant carcinoma was rarely observed with distal gastrectomy with Roux-en-Y operatives [6, 7]. This leads to the possibility that gastric remnant carcinoma may be induced by an exposure of the lower duodenal contents. Recent reports [8–10] have revealed that the average interval between initial gastrectomy and diagnosis of remnant cancer is usually more than 20 years, which is similar to the interval in our study. At present, there is no consensus regarding the time to begin surveillance, although annual upper gastrointestinal endoscopic examinations have been suggested [11].

Most patients with gastric remnant carcinoma are diagnosed during the symptomatic period with the cancer in the advanced stages, when the resectability rate is low and increased risk of comorbidities is present, as in the 4 patients with cardiovascular disease in our study. This emphasizes the importance of early diagnosis of gastric remnant carcinoma. Tumor location at the remnant site is possibly a prognostic factor; however, the variables between operated and unoperated patients in our study were not significantly related to the tumor location (*P* > 0.05), which is similar to that reported by Firat et al. [6] The ability to perform curative resection appears to be an important prognostic factor [12] becuse even after intensive radical gastrectomy, the patients had a favorable prognosis and good quality of life.

In conclusion, the incidence of gastric remnant carcinoma has not decreased, detecting gastric remnant carcinoma early, long-term follow-up, and an appropriate curative resection are important for long-term survival after surgery. As seen in the patients in this study, comorbidities such as myocardial infarction, cardiac arrhythmia, and uremia must also be prevented.

## Figures and Tables

**Table 1:** Presenting symptoms of the 21 patients with gastric remnant carcinoma.

Symptoms	Patients
Epigastric pain/fullness	9
Tarry stools/hematemesis	5
Poor appetite	4
Vomiting	3

**Table 2:** Clinicopathological features of the 9 patients with gastric remnant carcinoma who survived for over 2 years.

No.	Gender	Age (years)	Interval (months)	TNM staging	Anastomotic site^†^	Comorbidity	Previous surgery	Prognosis (years)
1	F	71	30	T1N3M0	**+**	Total gastrectomy	Billroth II	>4
2	F	52	20	T1N0M0	**+**	Total gastrectomy	Billroth II	>2
3	M	63	40	T1N0M0	**+**	Total gastrectomy	Billroth I	>2
4	M	72	16	T1N0M0	**+**	Total gastrectomy	Billroth II	>2
5	M	73	20	T1N1M0	**+**	Total gastrectomy	Billroth II	>2
6	M	67	32	T1N1M0	**+**	Chemotherapy	Billroth II	>2
7	F	62	40	T1N1M0	**+**	Total gastrectomy	Billroth II	>2
8	M	45	20	T1N1M0	**+**	Total gastrectomy	Billroth II	>2
9	M	80	50	T1N1M0	**+**	Total gastrectomy	Billroth II	>3

^†^Abbreviation: TNM: tumor lymph node metastasis.

**Table 3:** Clinicopathological features of the 12 patients with gastric remnant carcinoma who survived for less than a year.

No.	Gender	Age (years)	Interval (months)	TNM staging	Anastomotic site^†^	Comorbidity	Previous surgery	Prognosis (months)
1	M	73	35	T1N3M0		HCC	Billroth II	6
2	M	55	28	T1N0M1	**+**	Liver metastasis	Billroth II	10
3	M	80	55	T1N0M1	**+**	Intestinal metastasis	Billroth II	5
4	M	50	20	T1N1M0	**+**	LN metastasis	Billroth II	11
5	M	48	15	T1N1M1	**+**	Liver and LN metastases	Billroth II	4
6	F	60	30	T1N0M2	**−**	Pulmonary and cervical metastases	Billroth II	3
7	M	83	42	T1N0M0	**+**	Uremia on dialysis and hypertension	Billroth II	1
8	F	68	43	T1N1M0	**−**	LN metastasis (bypass)	Billroth II	2
9	M	77	16	T1N1M0	**−**	CVA and hypertension	Billroth II	11
10	M	58	30	T1N2M0	**+**	LN metastasis (palliative gastrectomy)	Billroth II	8
11	F	70	30	T1N1M0	**+**	LN metastasis	Billroth I	3
12	M	85	30	T1N1M0	**+**	AMI and LN metastasis	Billroth II	11

^†^Abbreviations: TNM: tumor-lymph node-metastasis; HCC: hepatocellular carcinoma; LN: lymph node; CVA: cerebrovascular accident; AMI: acute myocardial infarction.
